# Computer‐Assisted Implant Surgery: Patients' Experience and Perspectives

**DOI:** 10.1002/cre2.70143

**Published:** 2025-05-26

**Authors:** Xin Hui Yeo, Lin Jing Uei, Man Yi, Kajorn Kungsadalpipob, Keskanya Subbalehka, Bilal Al‐Nawas, Nikos Mattheos

**Affiliations:** ^1^ Department of Oral and Maxillofacial Surgery, Faculty of Dentistry Chulalongkorn University Bangkok Thailand; ^2^ Department of Stomotology Ditmanson Medical Foundation Chia‐Yi Christian Hospital Chia‐Yi Taiwan; ^3^ State Key Laboratory of Oral Diseases & National Center for Stomatology & National Clinical Research Center for Oral Diseases, West China Hospital of Stomatology Sichuan University Chengdu Sichuan China; ^4^ Department of Periodontology, Faculty of Dentistry Chulalongkorn University Bangkok Thailand; ^5^ Digital Implant Surgery Research Unit, Faculty of Dentistry Chulalongkorn University Bangkok Thailand; ^6^ Department of Oral and Maxillofacial Surgery University Medical Center Mainz Mainz Germany; ^7^ Department of Dental Medicine Karolinska Institute Stockholm Sweden

**Keywords:** CAIS, dental implants, guided surgery, patient‐reported outcomes, PREM, PROM

## Abstract

**Objectives:**

Although computer‐assisted implant surgery (CAIS) has increased significantly the precision of dental implant placement, documentation of the impact of such technologies in the patient‐reported experience and outcomes remains, however, limited. The aim of this white paper was to assess the impact of CAIS on key aspects of the patient experience, such as its potential benefits on (1) patients' understanding and engagement with implant surgery, (2) patient's confidence with treatment outcomes, (3) patients' preferences, (4) intra‐ and (5) Postoperative experience and (6) long‐term patient‐reported outcomes and oral health‐related quality of life.

**Material and Methods:**

A review of the literature compiled existing evidence from clinical studies up to November 2024, which was later discussed and synthesized with expert opinions and the best currently documented experience and practice.

**Results:**

No evidence was found that CAIS improves patient engagement or confidence with treatment outcomes, while comparative studies showed no difference in the intra‐ and postoperative experience when CAIS is used. Impact of the cost of CAIS procedures on patients perceptions and preferences has also not been explored, with the majority of studies significantly subsidizing patient costs, in particular randomized trials. At the same time, studies that compare interventions cannot assess the overall benefits of a complex workflow such as immediacy or minimally invasive approaches, to which CAIS is an essential part.

**Conclusions:**

Research on patient outcomes with CAIS might not fully reflect the potential of these technologies when limited to the level of the surgical intervention. Major anticipated benefits of CAIS for the patient such as the potential to reduce complexity and facilitate faster, safer and more predictable execution of digitally designed treatments, could be better approached in the future by studies aimed at assessing patient‐reported outcomes from entire treatment workflows.

## 1 Introduction

As computer‐assisted implant surgery (CAIS) systems become more widely adopted, the accuracy of dental implant placement using these technologies has been extensively studied and documented (Mahardawi et al. 2025; Zhou et al. 2025). However, the importance of other clinically relevant outcomes is being recognized and research is emerging (Sadilina et al. in press). To fully understand the future role of CAIS in implant practice, assessing patient‐reported outcomes (PROs) and experience (PRE) will be essential to provide a critical dimension that complements existing technical evaluations.

PROs and PRE play an increasingly vital role in monitoring the quality and effectiveness of patient‐centered care in implant dentistry. Validated instruments such as patient‐reported outcome measures (PROMs) and patient‐reported experience measures (PREMs) have been developed to streamline data collection and facilitate research advancement in these areas. An ITI Consensus Report published in 2018 was dedicated to patient‐reported outcome measures associated with implant dentistry (Feine et al. 2018). With an understanding of patient‐centered results, clinicians can support patients' perception of need, consider patient preference in clinical decision‐making, and improve clinician‐patient communication and quality of care (Fu et al. 2023).

Although PROs and PREs are currently considered essential parameters of clinical research (Calvert et al. 2013), their implementation is slow and not without challenges in most disciplines. Meaningful assessment of patient outcomes would require appropriate instruments, which might differ depending on the domain and anticipated impact of the assessed interventions. Wider, more extensive or long‐term treatment interventions might be best assessed by their impact on Oral Health‐Related Quality of Life (OHrQoL), while shorter, incremental interventions, protocols, or devices might require tailor‐made instruments, scales, and questionnaires to accurately reflect the patients' experience. CAIS interventions, predominantly belonging to the latter kind, have been scarcely assessed from the patients' perspective and with outcome measures of significant heterogeneity (Zhou et al. 2025). Data assessing patient experience can be difficult to interpret and generalize due to heterogeneity, as they tend to vary widely depending on individual circumstances, characteristics, and expectations (Yao et al. 2017). Furthermore, data of patient experience with CAIS interventions often lack valid comparisons, not only within the different CAIS systems, but also with conventional freehand protocols. As a result, there is currently limited evidence to determine if improved precision and accuracy translate to better patient experience and reported outcomes, including quality of life indicators and patient satisfaction (Pimkhaokham et al. 2022).

Assessing the currently available PROs and PREs data with CAIS protocols would supplement our understanding of these technologies with an essential insight into patients' attitudes, perceptions, priorities, motivation, as well as the impact of side effects and potential complications. At the same time, as PROs are scarcely assessed as primary outcomes in research within CAIS, collective assessment of the currently available literature would require extraction and qualitative synthesis of patient‐reported parameters from different studies.

The aim of this paper is to identify and compile published evidence of PROs and PREs with CAIS protocols and technologies and synthesize where possible with best practice and expert opinions, to offer deeper insights into patients' perceptions, attitudes and overall experience with the use of CAIS.

## 2 Methods

This white paper was based on a literature review conducted in major electronic databases aiming to identify studies assessing PROs or reporting findings related to patient experience and satisfaction with the use of CAIS. Clinical summaries or review articles were included and reviewed to identify relevant primary studies. Studies in English published up to 10 November 2024 were screened and relevant data extracted where available. There was no start date limitation for literature inclusion. Two of the co‐authors (X.H.Y. and L.J.U.) conducted the search, identification of studies, and data extraction. The number of identified studies investigating PROs, PREs, PROMs, or PREMs for CAIS is shown in Table 1.

**Table 1:** The number of studies investigating PROs, PREs, PROMs, or PREMs for CAIS (database search updated 10th November 2024).

CAIS modality	Number of studies on patient‐related reported outcomes	
	Primarily targeting PROMs	PROMs also reported
Static (s‐CAIS)	***n *= 10**	***n *= 15**
	Almahrous et al. (2020); Cristache et al. 2021; di Torresanto et al. 2014; Fortin et al. (2006); Joda et al. (2018); Kunavisarut et al. (2022); Marra et al. (2013); Nkenke et al. (2007); Sancho‐Puchades et al. (2019); Youk et al. (2014)	Abad‐Gallegos et al. (2011); Amorfini et al. (2017); Arısan et al. (2010); Komiyama et al. (2008); Lerner et al. (2020); Meloni et al. (2010); Merli et al. (2008); Nikzad et al. (2010); Özden Yüce et al. (2020); Penarrocha et al. (2012); Pomares (2010); Pozzi et al. (2014); Sannino and Barlattani (2016); Søndergaard et al. (2021); Tallarico et al. (2018); Van de Velde et al. (2010); Vercruyssen et al. (2014)
Dynamic (d‐CAIS)	***n *= 2**	***n *= 1**
	Nirula (2023); Zhu et al. (2024)	Jorba‐García et al. (2023)
Robotic (r‐CAIS)	*n *= 0	***n *= 1**
		Shi et al. (2024)
Static and dynamic combined (ds‐CAIS)	*n *= 0	***n *=1**
		Pomares‐Puig et al. (2023)
Comparative study between CAIS modalities	***n* = 2**	***n* = 1**
	Engkawong et al. (2021); Fu et al. (2023)	Heng et al. (2024)

The findings of the literature review were discussed and further synthesized with a group of expert clinicians with extensive clinical and research experience in the application of CAIS. The results and discussions focused on six main domains with clinical relevance, which were then organized and presented under the six main questions discussed in this paper.

### 2.1 Does CAIS Improve Patients' Understanding and Engagement With Implant Surgery?

#### 2.1.1 Summary

There is no evidence that patients' understanding, engagement, and treatment acceptance is different with the use of CAIS protocols as opposed to conventional analog workflows. Although CAIS workflow will offer an array of digital tools and software, this is commonly not intended for patient education, unless specifically designed for this purpose. Increasing patient engagement would require specific targeted communication and interventions, regardless of the use of CAIS or not.

#### 2.1.2 Explainer

**Figure 1:**
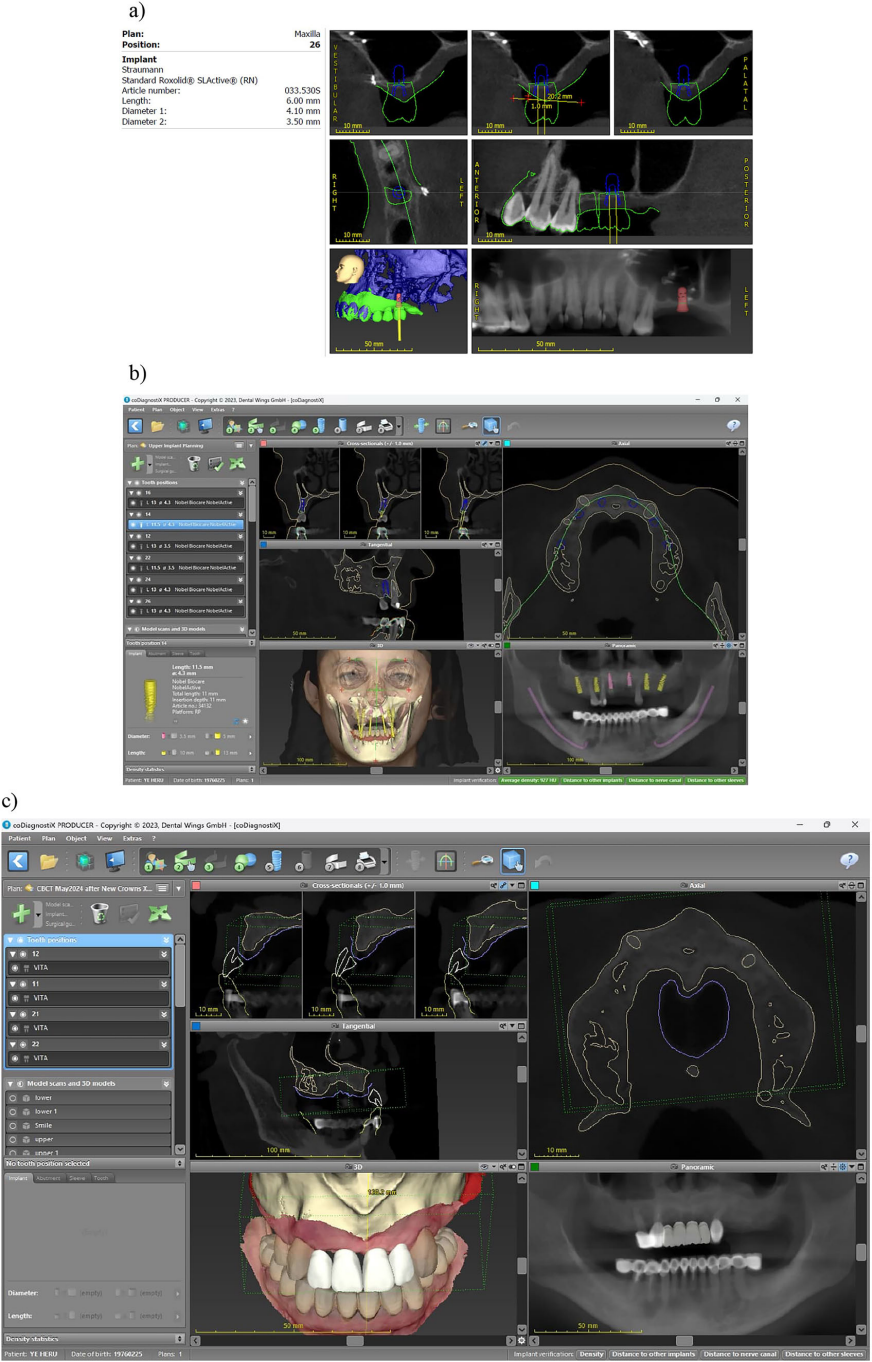
Typical visualizations from a CAD‐IPS intended to support planning for digital implant treatment plan. The visualizations and information provided are intended for trained clinicians and are not indicated as patient education material, who might find them difficult to understand or at times even intimidating. (a) Treatment plan export and (b, c) treatment plan tools on screen from CoDiagnostix, Dental Wings GmbH, Germany).

It is well established that patients' education and engagement is essential before initiation of implant therapy, and this process can be enhanced with visual aids and multimedia (Glaser et al. 2020). The use of CAIS will necessitate the use of 3‐dimensional planning, which reportedly could also be used for patient communication and engagement. At the same time, it can be argued that visualization of patient's own anatomy is not essential for patients' understanding of the procedure they are about to undergo. The computer‐aided design implant planning software (CAD‐IPS) and other software used during the CAIS workflow are not designed as patient education tools, although they could be used as such in certain cases (Figure 1a,b). The information and visualization offered by CAD‐IPS might be confusing, difficult to comprehend or even overwhelming to untrained eyes as most patients are. One could argue that generic, patient‐friendly multimedia and audiovisual animations could be equally or more engaging, effective and less intimidating to help patients understand the procedures and offer informed consent (Glaser et al. 2020).

**Figure 2:**
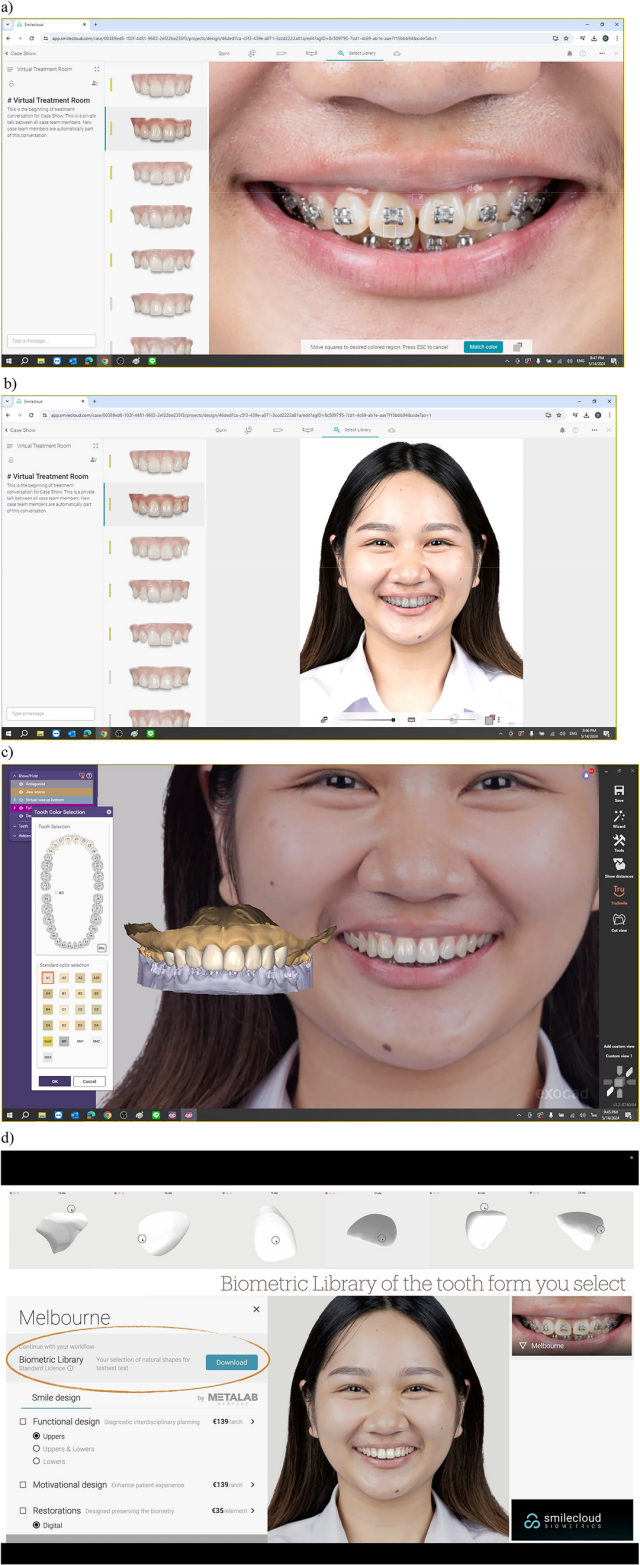
Digital Smile Design visualization simulating treatment outcome, suitable for patient education and communication purposes. (a–d) Different steps of simulating clinical outcome (right) after the selection of digital smile library (left). Patient's actual photo is used to simulate the outcome with different smile libraries, with the help of the uploaded 3D data of the patient and artificial intelligence algorithms. Photo courtesy: Dr. Jaijam Suwanwela, Implants and Esthetics, Chulalongkorn University, Bangkok, Thailand. Screenshot from Smile Cloud Biometrics, Romania.

On the other hand, CAIS has the potential to enhance our toolkit for efficiently promoting patient engagement and collaborative, personalized, patient‐centered care, especially, when the software utilized offers relatable, patient‐friendly visualization such as smile design and artificial intelligence (AI) powered simulated outcomes (Figure 2). It could be particularly appreciated by patients who wish in‐depth information about their treatment, possibly leading to better rapport between the anxious or curious patient and the treating clinician and improved patient satisfaction. Although simulated outcome visualization is available in certain workflows, it is not typically included in standard CAD‐IPS packages and may need to be arranged through additional software or modules. Recent development in AI and automated‐design features are gradually making these visualization tools more accessible and user friendly, potentially enhancing their role in patient communication. However, the effectiveness of these tools in improving patient understanding still needs to be systematically evaluated. It remains evident that patient engagement requires primarily the establishment of a therapeutic alliance with the patient (Pinto et al. 2012), which depends more on our ability to commit time and effort for face‐to‐face patient‐to‐doctor open‐ended communication, rather than the actual technology that will be used for the planning and execution of the surgery (Arunyanak et al. 2024).

### 2.2 Does the Use of CAIS Improve Patients' Confidence With Implant Surgery Outcomes?

#### 2.2.1 Summary

Although it is often argued that the use of CAIS could increase patients' confidence with treatment outcomes, there is no evidence supporting this. Patients' confidence as recorded retrospectively in some studies might be prone to bias under the novelty effect.

#### 2.2.2 Explainer

In conventional implant surgery, factors such as the surgeon's experience and three‐dimensional spatial awareness, human factors and errors (Chen et al. 2023), as well as limited access or visibility can affect the final implant positioning accuracy, which may lead to esthetic, biological and prosthetic complications (Chen et al. 2015). CAIS incorporates the use of CBCT diagnostic and virtual implant planning, which could enhance patient trust by demonstrating thorough preparation on the surgeon's part. This digital workflow provides better visualization of the planned treatment, potentially increasing transparency in the treatment planning process. This might explain the results of a study, where 10 patients with edentulous arches expressed confidence retrospectively that CAIS enhanced the outcomes of their surgical procedure (Pomares‐Puig et al. 2023). Despite no difference in overall satisfaction, the majority of patients scheduled for static computer‐guided implant surgery (s‐CAIS) by Amorfini et al. expressed significantly higher confidence in the procedure compared to those scheduled for traditional rehabilitation (Amorfini et al. 2017). Still, such an expression of confidence might be prone to institutional bias, social media impact on public perception or novelty effect associated with new technology, while it is often interrelated with confidence to the surgeon rather than the method/technology utilzed.

### 2.3 Do Patients Prefer the Use of CAIS Over Conventional Implant Surgery?

#### 2.3.1 Summary

When presented with a choice, patients appeared to strongly favor CAIS over conventional surgery, a choice which however might be subjected to a significant “novelty effect,” or greatly dependent on how CAIS is presented to the patient. On the other side, increased cost appears to be a barrier for the acceptance of CAIS by the patients, an effect that is however scarcely assessed.

#### 2.3.2 Explainer

Sancho‐Puchades et al. found that when asked preoperatively, the majority (83%) of patients favored the CAIS approach even without having prior experience of implant surgery (Sancho‐Puchades et al. 2019). Although most patients favored CAIS in the above study, their actual expectations of the surgery, as well as presurgery stress levels, did not differ from those who preferred conventional surgery (Sancho‐Puchades et al. 2019). This might suggest that patient preference for CAIS could be largely attributed to the “novelty effect,” often seen with new technology or innovation (Elston 2021). Such a novelty effect might have a stronger influence when patients are asked retrospectively to recall treatment‐related experience often long after treatment completion using satisfaction questionnaires (Diaz Abrahan et al. 2020). Patients with favorable attitudes toward AI, computerized care, and robotics might have the tendency to express higher satisfaction with CAIS (Nirula 2023). Interestingly, Lukkanasomboon et al. found in a recent clinical trial that the strongest factor influencing the decision of patients to opt for guided CAIS were the dentists' recommendation, while the strongest factors for those who opted for non‐guided CAIS was the cost (Lukkanasomboon et al. 2025).

There is very little reported with regard to the motivation and perceptions of patients who have volunteered for robotic CAIS (r‐CAIS) procedures, while it is understood that in most cases these are highly selected volunteers. The use of a display in dynamic and robotic CAIS could allow the surgeon to maintain a greater distance from patient's mouth which could reduce the risk of cross‐contamination (Pomares‐Puig et al. 2023) (e.g., in connection with potential COVID‐19 or other conditions), while it has been hypothesized that certain categories of patients such as patients with posttraumatic stress disorder (PTSD), phobia or autism may prefer their personal space not being invaded when they are in a vulnerable position (Raja et al. 2014). However, hypothesized on the contrary, the lack of proximity with the surgeon may increase anxiety in other categories of patients. The fact remains, however, that none of the currently available CAIS robots can fully replace the surgeon, as robots can only execute some procedures such as osteotomy and implant placement, while other procedures, like flap elevation and suturing, can only be conducted by the surgeon. The majority (96.6%) of patients felt comfortable with dynamic CAIS (d‐CAIS) even if the surgeon was not looking directly at the operating site (Nirula 2023).

When retrospectively assessing patients' experience, Youk et al. showed that s‐CAIS patients reported less discomfort and emotional distress (anxiety) and higher satisfaction compared to conventional surgery, but brought up the issue of financial burden associated with the cost of CAIS (Youk et al. 2014). Younes et al. argued that the higher costs involved with guided implant surgery are acceptable and clinically justified to guarantee a prosthetic‐driven outcome (Younes et al. 2019) from a clinician's perspective, but the patient's perspective is unknown.

When examining patient preference toward CAIS, it would be interesting to consider analysis of demographic characteristics such as patients' educational level and age which could be linked to preference for digitalized care.

#### 2.3.3 Critique

It is important to note that the majority of the studies comparing conventional surgery with CAIS (in particular the randomized trials) are conducted in universities and institutions which do not pass the additional costs of CAIS to the patients, thus neutralizing a possibly important factor from the patients' perspective, or even potentially favouring CAIS by reinforcing the perception of receiving a costly and innovative service at reduced costs. The majority of the non‐comparative studies did not specify if the patients' treatment costs have been subsidized and to what extent. It is very difficult to design randomized trials between different CAIS modalities which at the same time account for patients' perceptions and respect their choices or preferences, while simply recording the patients' preference prior to surgery might be subjected to novelty bias and the way treatments are presented by the surgeon.

### 2.4 Does CAIS Improve the Intraoperative Experience of the Patient?

#### 2.4.1 Summary

Patients' intraoperative experience with and without CAIS has not been directly compared. In general, patients' intraoperative experience appears closely linked to factors such as surgical duration and invasiveness, rather than the specific CAIS modality. There is limited data to determine which CAIS modality provides the best intraoperative experience, but an overall comparison might be not meaningful, as each CAIS modality has its own scope of indications, advantages and limitations.

#### 2.4.2 Explainer

Few studies that assessed intraoperative pain found no statistically significant difference in patients operated with s‐CAIS and conventional surgery (Sancho‐Puchades et al. 2019; Almahrous et al. 2020; Søndergaard et al. 2021). However, these studies involved a mixture of flapped and flapless surgeries. Fu et al. compared the use of s‐CAIS and d‐CAIS in fully edentulous patients (Fu et al. 2023) and found similar levels for anxiety, pain and mouth‐opening fatigue for all patients. It is reasonable to assume, however, that the scope of indications for each modality may exclude certain patients from a fair comparison. For example, to prepare an implant osteotomy at the angle dictated by the surgical guide into the distal part of the jaw using a guided drill, at least 4 cm of opening capacity is necessary (Amorfini et al. 2017). In difficult‐to‐access areas, d‐CAIS may offer an advantage by allowing tracking of the implant in a navigation screen, without relying on direct visualization in the patient's mouth (Block and Emery 2016). Likewise, eliminating the need for a surgical guide might help patients with exaggerated gag reflex, as foreign body sensation is likely to trigger a gag reflex and interfere with the surgical process (Sakamoto et al. 2016). Although mostly well tolerated, a surgical guide is more frequently associated with foreign body sensation (Fu et al. 2023), pharyngeal reflex (Fu et al. 2023), and discomfort (Søndergaard et al. 2021) during surgery than those treated with d‐CAIS. There was no significant difference reported between the marker‐free and the marker‐based groups regarding the intraoperative discomfort caused by keeping the mouth open and the presence of d‐CAIS devices (Zhu et al. 2024). The r‐CAIS literature currently lacks data on patients' intraoperative experience, their intrinsic motivation as well as perceptions and attitudes toward these technologies, which highlights an important void in the literature.

Duration of the surgical intervention is an important measure of efficacy as well as an important parameter of patient experience. It also correlates with the frequency and intensity of postsurgical pain and healing complications (Pimkhaokham et al. 2022; Sancho‐Puchades et al. 2019). Limited evidence suggests that s‐CAIS significantly reduced the duration of complex interventions such as multiple implant placement in **fully edentulous** patients, particularly using flapless surgical technique (Nocini et al. 2013; Arısan et al. 2010) and also in lateral window osteotomy during sinus augmentation (Narongchai et al. Forthcoming). In guided lateral window sinus augmentation, the use of a surgical stent can reduce the time required for the lateral window osteotomy, while helping surgeons avoid certain anatomical landmarks such as septa and vessels. In the partially edentulous patients, Amorfini et al. (2017) and Younes et al. (2019) showed a significantly reduced surgical duration for fully‐guided and pilot‐guided s‐CAIS implant placement compared with non‐guided placement, whereas Pozzi et al. (2014) and Schneider et al. (2019) reported similar surgical procedure duration between s‐CAIS and non‐guided or conventional surgery. However, in cases of single edentulous space, non‐guided CAIS or conventional surgery appeared consistently faster in the absence of complexity (Pimkhaokham et al. 2022; Engkawong et al. 2021; Kaewsiri et al. 2019) as the use of CAIS is not without potential delays: deficient fit and instability of the surgical guide requiring intraoperative adjustments can add to the treatment duration of s‐CAIS (Mangano et al. 2018). d‐CAIS, on the other hand, eliminates the need of a surgical guide and thus the risk of intraoperative adjustments, however, the multiple registration procedures can add to intraoperative or overall treatment time and perceived treatment complexity. Jorba‐García et al. found d‐CAIS surgeries placing mainly 1–2 implants on partially dentate patients lasted on average 14 min longer than corresponding freehand surgeries (Jorba‐García et al. 2023), with all patients however assessing the duration of the surgeries as “acceptable” (Jorba‐García et al. 2023). Likewise, the reduction of the average duration of surgery from 69 to 56 min when s‐CAIS was used for sinus augmentation, did not lead to any significant improvements in PROMs (Narongchai et al. Forthcoming). Conclusively, there appears to be inconsistent results with regard to the potential of s‐CAIS and d‐CAIS to reduce the duration of surgeries, while any potential benefits appear more likely to be manifested in procedures with increased complexity.

With regard to r‐CAIS, estimating the intraoperative time might be even more complex, but remains essential to reflect patient's in‐the‐chair time and intraoperative comfort. Apart from the obvious complexity of combining surgical procedures conducted by the surgeon (e.g., reflection of the flap, suturing, etc.) with procedures conducted by the robot (osteotomy, implant placement), intraoperative procedures between different CAIS robots might different significantly depending on their level of autonomy (Xu et al. 2023). Furthermore, essential registration procedures at different stages might be time consuming and are conducted differently in different CAIS robots. A report based on a semi‐active robot quoted surgery duration to be 20–25 min in 19 single‐implant surgery cases and 47 and 70 min for 2 edentulous arch surgeries, which the authors found comparable to freehand surgeries (Qiao et al. 2023). The overall intraoperative time with r‐CAIS is at present difficult to objectively assess due to the diversity of workflows and devices as well as the scarcity of data, but based on the expert reports, it is reasonable to assume that it will easily exceed that of freehand surgery. The time efficiency of d‐CAIS and r‐CAIS might be apparent in cases of multiple implants placement (Qiao et al. 2023). Operator proficiency and the learning curve associated with CAIS may also have an impact on the patient experience.

#### 2.4.3 Critique

Patients' intraoperative experience when CAIS is used has been approached in research mainly through surrogate measures such as procedure duration, yielding inconsistent results, albeit with a trend to favor CAIS in procedures of high complexity (e.g. full arch procedures). Given the diversity of procedures and techniques utilized under CAIS, location and number of implants placed and different levels of invasiveness, scarce data reporting Postoperative PREs cannot support any general conclusions. The extent of the invasiveness of the surgery (e.g., flapped or flapless procedure) might significantly influence the patients' experience, but whether it links to the use of CAIS or not has not been systematically assessed.

### 2.5 Does the Use of CAIS Improve Post‐Surgery Healing Experience and Outcomes?

#### 2.5.1 Summary

Comparative studies have not found any difference in postoperative outcomes related to patients' healing experience between CAIS and conventional implant surgery. However, in a field where choice of technology should be driven by the indications, comparative studies and in particular randomization might be not the optimal instrument to investigate patients' experience due to inherent limitations.

#### 2.5.2 Explainer

Postoperative pain and swelling are the most common sources of morbidities after implant placement. A meta‐analysis of a few comparative studies (Sancho‐Puchades et al. 2019; Engkawong et al. 2021; Kunavisarut et al. 2022) in 2021 showed no statistical difference in postoperative healing (pain, swelling, bruising, bleeding) events and functional disturbances reported by patients between CAIS used with flap elevation and conventional implant placement (Pimkhaokham et al. 2022). A comparative study on postoperative healing PROs reported mild oral health‐related impairment in OHIP‐5 for both the marker‐based and marker‐free d‐CAIS groups (Zhu et al. 2024) after implant placement under a flap. Pomares‐Puig et al. observed no postoperative complications, such as pain, inflammation, or hematoma, and also no phonetic, esthetic, or chewing ability problems with the combined ds‐CAIS technique (Pomares‐Puig et al. 2023).

One of the reported advantages of CAIS is reduced invasiveness of surgical procedures and thus also minimizing the discomfort and swelling (Pozzi et al. 2014; Joda et al. 2018). CAIS allows for flapless or limited flap elevation, resulting in less postoperative morbidity to the patient. Flapless surgery is linked to reduced pain, less analgesic consumption, less swelling, shorter chair‐time and reduced risk of hemorrhage while achieving greater patient satisfaction (Gargallo‐Albiol et al. 2019). Engkawong et al. noted that the greatest postoperative swelling on the second day had a significant correlation with flap operation, although only four cases in the study utilized flapless surgery (Engkawong et al. 2021). Fortin et al. demonstrated that patients who underwent a flapless procedure combined with s‐CAIS required significantly fewer postoperative analgesics compared to those who had conventional freehand open flap surgery (Fortin et al. 2006). Similarly, Arisan et al. reported that, within the s‐CAIS technique, patients who received flapless s‐CAIS consumed fewer analgesics postoperatively compared to those who underwent an open flap procedure (Arısan et al. 2010). Other similar comparative studies which used flapless technique for s‐CAIS and flapped technique for conventional surgery showed significant differences in the postoperative pain score (Arısan et al. 2010; Fortin et al. 2006; Vercruyssen et al. 2014; Nkenke et al. 2007). The same was also true for zygomatic implant surgeries assisted by d‐CAIS, where the flapless group exhibited significantly lower postoperative pain and swelling compared to the group who underwent flapped surgery (Bhalerao et al. 2023). On the contrary, Jorba‐García et al. found that when both d‐CAIS and conventional surgery were conducted mainly flapless, no difference was shown in 7‐day postoperative pain and analgesic intake (Jorba‐García et al. 2023). Synthesizing all the above, it is apparent that flapless surgery is a more potent determinant of postoperative pain, swelling, and OHrQoL (Pimkhaokham et al. 2022) than which CAIS modality was used.

Another reported advantage of CAIS comes from its potential role in facilitating immediacy: both in terms of placement in fresh extraction socket but also with regard to temporary or final prosthetic restoration with prefabricated prosthesis. The time required to fit the temporary prosthesis was found to be significantly shorter in s‐CAIS than the freehand group, directly ascribed to the greater surgical precision and more accurate prosthesis fabrication afforded by CAIS (Amorfini et al. 2017). Patient satisfaction with the overall treatment is shown to be more affected by the presence of immediate restoration than postoperative discomfort (Amorfini et al. 2017; Van de Velde et al. 2010), particularly in the esthetic zone, where patient's expectations of rehabilitation are increased.

#### 2.5.3 Critique

There is a major limitation of current research in postoperative PROs with the use of CAIS, as comparative studies are focused on procedures, while the main benefits of CAIS from the patient perspective derive from CAIS empowering specific overall treatment workflows and outcomes. In other words, when a flap is raised, osteotomy is being conducted and an implant placed, there is little reason to suggest any difference in postoperative PROs as a result of the procedure being conducted with or without CAIS. At the same time, CAIS can become a powerful tool to the extent that its use empowers treatment workflows such as immediate implant placement, immediate provisionalization and/or loading, procedures with documented major impact on patient experience and satisfaction. Unfortunately, there are no comparative studies at the workflow level, a task that would be difficult to design and execute, but certainly a worthy aim for future research.

### 2.6 Does CAIS Improve Long‐Term Patient‐Reported Outcomes and OHrQoL?

#### 2.6.1 Summary

Very few studies have investigated long‐term PROs following CAIS. Again, the long‐term patient benefits of CAIS are more likely to derive from its ability to empower overall patient‐centered workflows such as restorative driven treatment planning and rather than use of CAIS in isolated, independent procedures.

#### 2.6.2 Explainer

OHRQoL is a multidimensional construct that includes a subjective evaluation of the individual's oral health, functional well‐being, emotional well‐being, expectations and satisfaction with care, and sense of self (Sischo and Broder 2011). OHrQoL includes post‐surgery symptoms, function disturbance, including the ability to perform oral hygiene, chew, and speak. OHrQoL is a broad assessment tool designed to measure the overall impact of a disease or extensive treatment on a patient's life. Thus, OHRQoL scales may not specifically capture nuances relevant to relatively short interventions such as CAIS, which could benefit from more targeted assessment tools designed to reflect patient experience specific to this technology. Long‐term OHRQoL scores are more reflective of the impact of implant‐supported or assisted prostheses (Nickenig et al. 2016) rather than the mode of implant surgery.

Only few studies on CAIS reported on patient satisfaction or PROs beyond 1 year (Meloni et al. 2010; Heng et al. 2024; Tallarico et al. 2018). All other studies which assessed OHrQoL after CAIS surgery only did so immediately after surgery, for the first week (Jorba‐García et al. 2023; Kunavisarut et al. 2022; Vercruyssen et al. 2014) and up to 2 weeks post‐surgery (Schneider et al. 2019). Meloni's study interviewed patients at 18 months post‐surgery, and 13 out of 15 patients reported that their quality of life and lifestyle improved with the implant‐supported maxillary prosthesis and considered the s‐CAIS surgery worthwhile (Meloni et al. 2010).

With the increasing importance of restorative design for the long‐term sustainable health of implant therapy (Rungtanakiat et al. 2023; Janda and Mattheos 2024) when the implant, prosthesis, tissue, and biofilm are perceived as a system in close synergistic interaction (Mattheos et al. 2021; Pedrinaci et al. 2024), the role of CAIS as a key facilitator of design‐driven implant therapy becomes apparent. An implant placed by means of CAIS as part of an evidence‐based, design‐driven implant treatment plan, may reduce the number of biological and mechanical complications in the long‐term which could impact on quality of life, such as peri‑implant diseases, screw loosening or esthetic issues (Yogui et al. 2021). However, such effect might only be measurable in the long term and through carefully designed comparative studies. Heng et al. found the use of CAIS to lead to superior outcomes in terms of Pink Aesthetic Score and marginal bone loss compared to non‐guided placement for implants in the aesthetic zone in medium to long‐term follow‐up (Heng et al. 2024). Regardless, the difference in Pink Aesthetic Score (PES) was not reflected in patient‐reported satisfaction, which was not different between the patients who received the implants via CAIS and those via conventional methods.

Long‐term studies should also consider the economic impact of CAIS on patients, including potential savings from reduced complications and maintenance needs. This could provide valuable information for both clinicians and patients when weighing the initial higher costs of CAIS against potential long‐term benefits.

#### 2.6.3 Critique

Instruments developed to capture the impact of treatment interventions in the long term, such as the OHrQoL questionnaires, are much more suitable to assess wider treatment outcomes than specific and relatively short interventions such as implant surgery with or without CAIS. Given the increasing consensus of the patient benefits deriving from the evidence‐based design‐driven implant treatment, CAIS can contribute to long‐term sustainable success of implant therapy as a key link empowering such workflows.

## 3 Discussion

**Figure 3:**
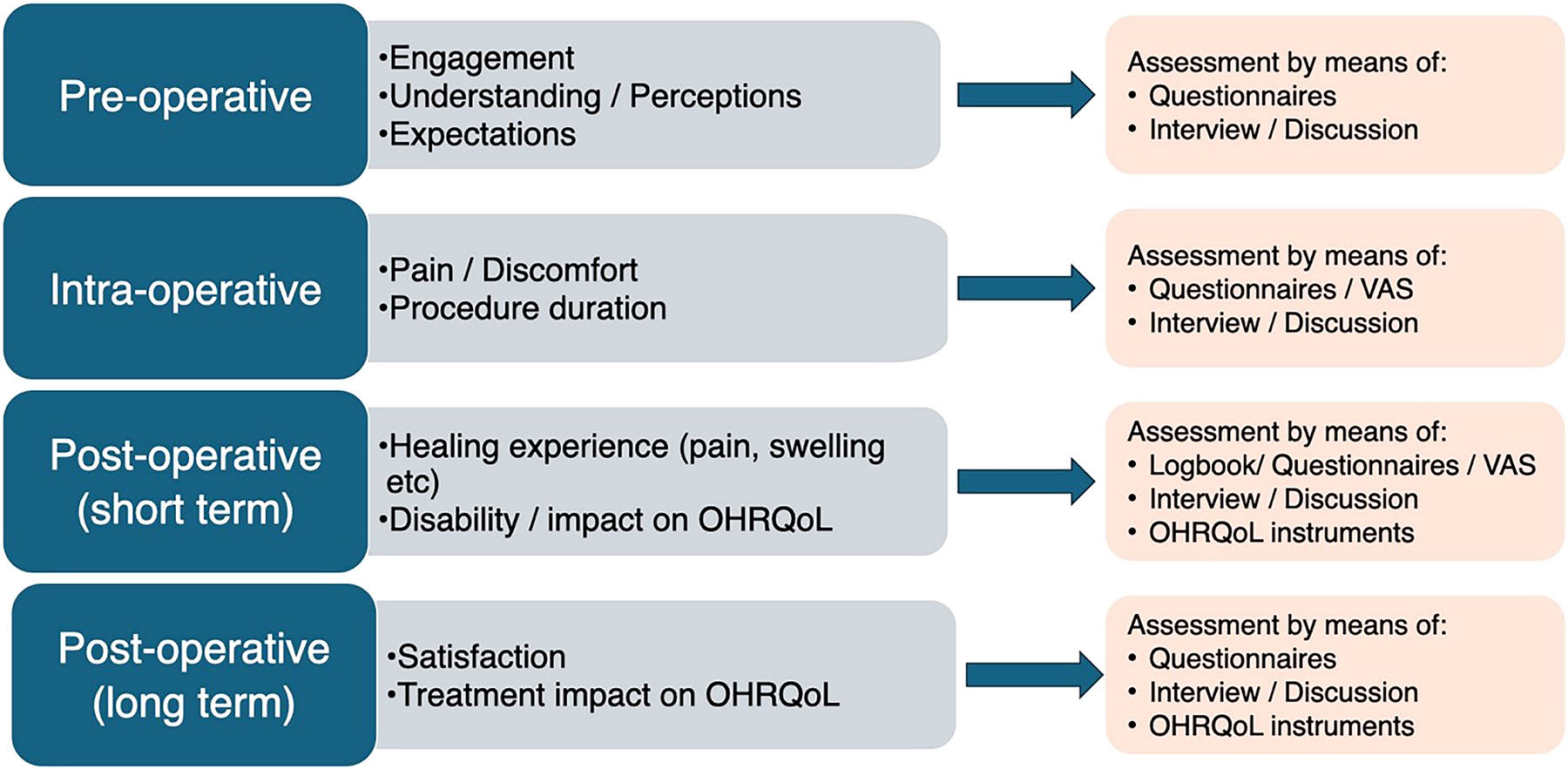
Illustration of important patient‐centered parameters at different stages of the treatment that can be targeted by specific assessments at intervention level by means of PROMs. Preoperative assessments can include patients' understanding and engagement, while intraoperative focus more on the actual experience with the intervention procedure. Assessments immediately, the 2–4 weeks following the surgical intervention, focus on the patients' experience with healing symptoms and potential impact on esthetics, function and overall well being and are again specific to the intervention level. Finally, long‐term postoperative assessments can assess the overall treatment of the treatment on quality of life and can document impact of the entire treatment/workflow level.

**Figure 4:**
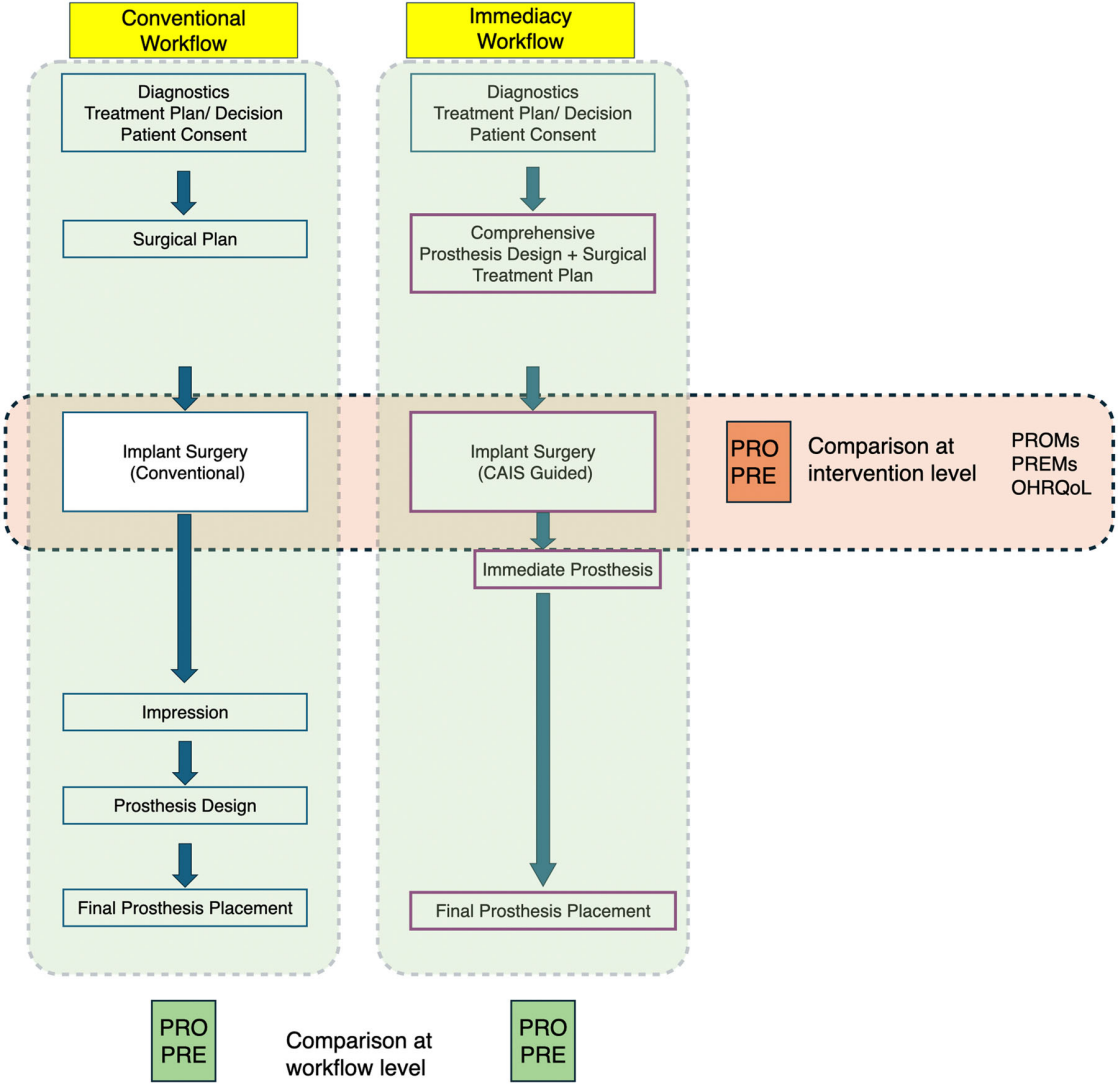
Illustration of the difference between comparisons at intervention (horizontal) and workflow (vertical) level. Patient‐reported outcomes and experience can be recorded at both levels. Currently available comparative studies and randomized trials focus at the intervention level only, typically showing no difference between CAIS and conventional surgeries. At the same time a major benefit of CAIS is that as an essential component, it can enable more patient friendly workflows which have potential to significantly increase patient benefits. Comparative studies assessing PROMs, however, at the Workflow level are not yet available.

The purpose of any clinical intervention is to serve the patients' interests not only as assessed in clinical outcomes, but also in tangible improvements in patients' experience with the treatment and resulting quality of life. The use of CAIS has the potential to improve both, but the assessment of patients' perspective might not always be straightforward. There are two obvious levels where the use of CAIS can result in significant benefits: the intervention and the workflow (Figures 3 and 4). At the first level, we focus on the impact of the actual surgical procedure and its direct outcomes. On the second level, however, we see CAIS as a critical enabler of a wider and more complex workflow, with significant overall patient benefits.

### 3.1 Intervention‐Level Analysis

Most of the currently available comparative studies are focused on this level, the procedure, where little difference can be seen regarding PREs with CAIS or conventional surgery. It's hardly surprising that surgical interventions involving similar tissue manipulation, incisions, osteotomies, and suturing (as known to be essential in comparative studies) would yield similar intraoperative and healing experience, regardless of whether implant placement is guided. Furthermore, whether an implant is surgically placed as part of a design‐driven system or based on a “bone‐driven” paradigm may be critical for long‐term sustainable health, but the difference may not be reflected in any postoperative PROs or PREs assessed at the intervention level. Hence, the current scientific paradigm defines the digital treatment plan as a foundation of CAIS, with the aim to identify the 3‐dimensional patient‐optimized implant position (Jorba‐Garcia et al. 2025). Nevertheless, the long‐term benefits of prosthetically‐driven implant placement (of which workflow CAIS in an essential prerequisite) should be assessed in a systematic manner. This calls for standardized protocols and instruments specifically designed to assess patient experience with CAIS interventions. Current OHrQoL measures, while valuable for overall treatment assessment, may not capture the nuanced impacts of specific CAIS workflows. The development of such instruments should consider both direct experience with the procedures but also and long‐term outcomes.

### 3.2 Workflow‐Level Analysis

CAIS can be a valuable tool in an advanced workflow, but of limited benefit if the outcome is perceived only as an isolated surgical procedure. By empowering the precise execution of an evidence‐based and design‐driven presurgical plan, CAIS could significantly contribute to the longevity and success of implant therapy and thus greatly benefit the patient. By providing high precision in flapless surgery or extraction sockets and by empowering the use of prefabricated prosthesis for immediate use, CAIS can significantly transform patient experience and increase satisfaction. Such benefits, however, can only be assessed at the overall treatment level. Comparative studies could be much more challenging to design and conduct when the intention is to compare different and complex workflows rather than specific interventions. There is certainly a need for improved clinical studies assessing entire workflows, but at the same time a long list of ethical, sampling, and logistics limitations might make such studies extremely difficult, especially if the aim is to conduct randomized trials. Thus, to address the benefits of CAIS as a component of an advanced treatment sequence, a pragmatic approach is warranted, where clinical trials are combined with wider assessments of the literature supplemented with best clinical practice and experience.

It is often assumed that the use of CAIS will allow increased patient engagement and motivation, but this is certainly not self‐evident. There is little understanding in patients' intrinsic motivation in selecting CAIS technologies, and evidence suggests that the dentist's recommendation to be still the major influence. The essential tools for utilizing CAIS are designed for use by trained clinicians and do not necessarily include modules or components intended for patient education. Patient engagement remains a goal that requires significant effort, time, and dedicated tools, rather than an outcome depending on the technology used for the implant placement. Although the use of CAIS might improve clinicians' array of communication and engagement methods, the aim remains to establish a therapeutic alliance with the patient independently of the technology used.

Likewise, it is unclear whether the use of CAIS would increase patients' confidence with treatment. Quantifying patients' confidence with the outcomes of largely elective procedures such as implant surgery is not easy and the fact that the patients have already decided to proceed with the treatment reflects an already established confidence with the outcomes, which greatly depends on how the procedure has been presented and explained and is often interrelated with confidence on the operator/surgeon, especially if CAIS appears as the recommendation/preference of the surgeon (Axelrod 2000; Hamelin et al. 2012). How CAIS technologies can influence patients' confidence in the care they receive is a very important parameter, which could be objectively assessed in the future only with carefully designed studies at workflow level.

The cost of implementing CAIS in clinical practice and potential impact this might have on patients' perceptions and experience remains unknown. Much of our understanding on patients' experience comes from randomized clinical trials, where the costs of the different CAIS utilized were subsidized by the experiments, thus excluding a potentially very relevant parameter for clinical practice. In the future, deeper insights in the impact of costs and cost‐effectiveness will be required to optimize the use of CAIS in wider clinical settings.

## 4 Concluding Remarks

As CAIS technology continues to evolve, focusing on PROs and PREs remains crucial for its meaningful integration into clinical practice. Future developments should prioritize not just technical precision, but also patient comfort, cost‐effectiveness, and impacts toward long‐term treatment success. This patient‐centered approach, combined with sharing of best practice and rigorous clinical evidence, will help establish CAIS as an integral component of modern implant dentistry.

There can be significant benefits for the patient when CAIS is perceived as an essential component enabling a digital workflow which helps the patient achieve the treatment outcomes faster, safer, and with reduced invasiveness than conventional workflows. However, research has not been conducted on this level yet and the inherent limitations of randomized trials might make this goal very difficult to reach. On the other hand, the body of literature on CAIS includes assessments of PROs and PREs mostly at the intervention level, where typically little difference is shown. However, with allocation of patients to different technologies based on randomization and not indication‐driven, much of the actual potential of these technologies cannot be assessed. The growing attention to the patient perspective in research and clinical practice, including a dedicated ITI Consensus to PROMs (Feine et al. 2018), has highlighted the importance of patients' perspective in implant dentistry, encouraging the evaluation of the quality and success of care from the patient's viewpoint. At the same time, it becomes evident that existing approaches assessing PROMs and PREs have inherent limitations that often prevent capturing the role of these technologies in delivering patient‐centered treatments. Together with increased attention in PROs as a supplement of clinical research, a collective effort is required toward the development of comprehensive instruments and protocols to properly assess the patients' benefits from the use of CAIS. Bringing together different stakeholders, such as patients, clinicians, industry, and technology developers, in focus groups could help advance the field of digital implant dentistry in line with clinical needs and patient priorities.

## Author Contributions

*Conceptualization*: Bilal Al‐Nawas and Nikos Mattheos. *Data curation*: Xin Hui Yeo, Lin Jing Uei, Man Yi, Kajorn Kungsadalpipob, and Keskanya Subbalehka. *Formal analysis*: Xin Hui Yeo, Man Yi, and Nikos Mattheos. *Investigation*: Xin Hui Yeo and Lin Jing Uei. *Methodology*: Bilal Al‐Nawas and Nikos Mattheos. *Project administration*: Bilal Al‐Nawas and Nikos Mattheos. *Resources*: Bilal Al‐Nawas and Nikos Mattheos. *Supervision*: Bilal Al‐Nawas and Nikos Mattheos. *Validation*: Xin Hui Yeo, Lin Jing Uei, and Nikos Mattheos. *Visualization*: Xin Hui Yeo and Nikos Mattheos. *Writing – original draft*: Xin Hui Yeo and Nikos Mattheos. *Writing – review and editing*: Lin Jing Uei, Man Yi, Kajorn Kungsadalpipob, Keskanya Subbalehka, and Bilal Al‐Nawas.

## Conflicts of Interest

The authors declare no conflicts of interest.

## Data Availability

The authors have nothing to report.
